# Supplementary material for: Secondary Brain Injury After Parenchymal Cerebral Hemorrhage in Humans: The Role of NOX2-Mediated Oxidative Stress and Endothelin-1
Source: Int J Mol Sci. 2024 Dec 7;25(23):13180. doi: 10.3390/ijms252313180 (PMC11642479; doi:10.3390/ijms252313180)
Supplement: Supplementary file 1 [file ijms-25-13180-s001.zip › ijms-3310063-supplementary.pdf]

Supplementary Materials

Supplementary Table S1. Radiological characteristics of hemorrhagic stroke in all study patients and by presence/absence of ischemic lesions at T1 (statistically significant differences are in bold).

	**All patients**<br>**N=28**	**Ischemic lesions**<br>**n=12**	**No ischemic lesions**<br>**n=16**	**p value**
Angiography (%)<br>- no<br>- yes<br>- yes, pathologic	 14/18 (77.8)<br>3/18 (16.7)<br>1/18 (5.6)	 8/10 (80.0)<br>2/10 (20.0)<br>0	 6/8 (75.0)<br>1/8 (12.5)<br>1/8 (12.5)	0.493
Hematoma side (%)<br>- left<br>- right<br>- bilateral	 14/28 (50.0)<br>12/28 (42.9)<br>2/28 (7.1)	 4 (33.3)<br>7 (58.3)<br>1 (8.3)	 10 (62.5)<br>5 (31.3)<br>1 (6.3)	0.304
Hematoma localization (%)<br>- deep<br>- lobar	 13/27 (48.1)<br>14/27 (51.9)	 5 (41.7)<br>7 (58.3)	 8/15 (53.3)<br>7/15 (46.7)	0.704
T1				
Time of onset-CT at T1, h, median (IQR)	2.3 (1.5-3.2)	2.0 (1.4-5.7)	2.32 (1.47-3.50)	0.875
Time of onset-MRI at T1, h, median (IQR)	9.9 (3.3-21.8)	14.8 (4.8-20.3)	6.95 (3.23-22.70)	0.947
Hematoma volume at T1, median (IQR), cm3	11.9 (5.8-32.1)	23.05 (13.40-41.03)	6.65 (2.85-12.40)	**0.005**
Peri-hematoma edema at T1 (%)<br>- absent<br>- cortical sulci narrowing<br>- ventricles asymmetry<br>- midline shift	 2/28 (7.1)<br>3/28 (10.7)<br>10/28 (35.7)<br>13/28. (46.4)	 0<br>0<br>4 (33.3)<br>8 (66.7)	 2 (12.5)<br>3 (18.8)<br>6 (37.5)<br>5 (31.3)	0.131
Edema severity at T1 (%)<br>- Absent/mild/moderate<br>- Severe	 15/28 (53.6)<br>13/28 (46.4)	 4 (33.3)<br>8 (66.7)	 11 (68.8)<br>5 (31.3)	0.063
Peri-hematoma edema volume at T1, median (IQR), cm3	11.40 (4.13-19.33)	15.25 (11.05-27.78)	8.15 (3.85-12.20)	**0.046**
Midline shift at T1, mm, mean (SD)	2.59 (3.89)	4.45 (4.74)	1.31 (2.62)	**0.036**
Intraventricular hemorrhage at T1 (%)	14/28 (50.0)	8 (66.7)	6 (37.5)	0.127
Ischemic lesions at T1 (%)<br>- Not remote<br>- Remote	12/28 (42.9)<br>11/28 (39.3)<br>1/28 (3.6)	 11 (91.7)<br>1 (8.3)	-	-
Number of ischemic lesions a T1 (%)<br>- single	12/28 (42.9)<br>7/28 (25.0)	12 (100.0)<br>7 (58.3)	-	-

- multiple	5/28 (17.9)	5 (41.7)		
Remote ischemic lesions at T1 (%)<br>- ipsilateral<br>- controlateral	1/28 (3.6)<br>0<br>1/28 (3.6)	1 (8.3)<br>0<br>1 (8.3)	-	-
Ischemic lesions morphology at T1 (%)<br>- perihematomal<br>- not perihematomal	 6/12 (50.0)<br>6/12 (50.0)	 6/12 (50.0)<br>6/12 (50.0)	-	-
Island sign at T1 (%)	14/27 (51.9)	10 (83.3)	4/15 (26.7)	**0.003**
Hemispheric hypoperfusion at T1 (%)	5/26 (19.2)	4/10 (40.0)	1 (6.3)	0.055
Fazekas Scale (%)<br>- 0 (absent/very mild)<br>- 1 (mild)<br>- 2 (moderate)<br>- 3 (severe)	 3 (11.1)<br>10 (37.0)<br>8 (29.6)<br>6 (22.2)	 1/11 (9.1)<br>4/11 (36.4)<br>2/11 (18.2)<br>4/11 (36.4)	 2 (12.5)<br>6 (37.5)<br>6 (37.5)<br>2 (12.5)	0.464
Fazekas Scale (%)<br>- 0-1 (absent-mild)<br>- 2-3 (moderate-severe)	 13/27 (48.1)<br>14/27 (51.9)	 5/11 (45.5)<br>6/11 (54.5)	 8 (50.0)<br>8 (50.0)	0.816
Microbleeds (%)<br>- absent<br>- present	 19/28 (67.9)<br>9/28 (32.1)	 7 (58.3)<br>5 (41.7)	 12 (75.0)<br>4 (25.0)	0.432
Number of microbleeds (%)<br>- ≤10<br>- >10	 7/9 (77.8)<br>2/28 (22.8)	 4 (33.3)<br>1 (8.3)	 3 (18.8)<br>1 (6.3)	 0.387<br>0.835
T2				
Time of onset-MRI at T2, h, median (IQR)	67.1 (53.5-110.3)	59.2 (47.0-110.9)	75.71 (53.60-110.29)	0.753
Hematoma volume at T2, mean (IQR), cm3	10.20 (6.8-36.5)	19.55 (9.80-42.05)	6.90 (6.20-27.70)	**0.043**
Hemorrhage volume variation from T1 to T2, mean (IQR), cm3	-1.1 (-2.30, 3.25)	-0.65 (-4.45, 2.28)	-1.10 (-1.80, 3.70)	0.531
Hematoma volume increase (%)	5/17 (29.4)	2/8 (25.0)	3/9 (33.3)	1.0
Perihematomal edema at T2 (%)<br>- absent<br>- cerebral sulci<br>- ventricles asymmetry<br>- midline shift	 2/18 (11.1)<br>1/18 (5.6)<br>6/18 (33.3)<br>9/18 (50.0)	 0<br>0<br>1/8 (12.5)<br>7/8 (87.5)	 2/10 (20.0)<br>1/10 (10.0)<br>5/10 (50.0)<br>2/10 (20.0)	**0.040**<br>-<br>-<br>0.103<br>**0.006**
Edema severity at T2 (%)<br>- Absent/mild/severe<br>- Severe	 9/18 (50.0)<br>9/18 (50.0)	 1/8 (12.5)<br>7/8 (87.5)	 8/10 (80.0)<br>2/10 (20.0)	**0.015**
Perihematomal edema volume at T2, mean (IQR), cm3	23.50 (13.7-38.5)	38.0 (20.18-43.08)	14.80 (9.85-23.65)	**0.012**

Edema volume variation from T1 to T2, median (IQR), cm3	4.8 (1.2, 15.4)	12.50 (-2.03, 19.0)	4.3 (3.10, 13.20)	0.596
Edema volume increase (%)	13/16 (81.3%)	5/7 (71.4)	8/9 (88.9)	0.550
Midline shift at T2, mm, mean (SD)	2.94 (3.54)	4.75 (3.22)	1.3 (2.99)	**0.035**
Intraventricular hemorrhage at T2 (%)	6/17 (35.3)	3/7 (42.9)	3/10 (30.0)	0.644
Ischemic lesions at T2 (%)<br>- Not remote<br>- Remote	9/15 (60.0)<br>7/15 (46.7)<br>2/15	6/7 (85.7)<br>4/7 (57.1)<br>2/7 (28.6)	3/8 (37.5)<br>3/8 (37.5)<br>0	0.066<br>0.462<br>0.200
Number of ischemic lesions at T2 (%)<br>- single<br>- multiple	9/15 (60.0)<br>3/15 (20.0)<br>6/15 (40.0)	6/7 (85.6)<br>2/7 (28.6)<br>4/7 (57.1)	3/8 (37.5)<br>1/8 (12.5)<br>2/8 (25.0)	0.066<br>0.453<br>0.220
Remote ischemic lesions at T2 (%)<br>- ipsilateral<br>- controlateral	2/15 (13.3)<br>2/15 (13.3)<br>0	2/7 (28.6)<br>2/7 (28.6)<br>0	0	0.200
New ischemic lesions at T2 vs at T1 (%)	3/16 (18.8)	0	3/8 (37.5)	0.200
Ischemic lesions at T1 not visible at T2 (%)	2/16 (12.5)	2/8 (25.0)	0	0.467

IQR= interquartile range; SD= standard deviation.

Supplementary Table s2. Clinical outcome measures in all study patients and by presence/absence of ischemic lesions at T1.

	**All patients**<br>**N=28**	**Ischemic lesions**<br>**n=12**	**No ischemic lesions**<br>**n=16**	**p value**
mRS at 3 months (%)<br>- 0<br>- 1<br>- 2<br>- 3<br>- 4<br>- 5<br>- 6	 2/23 (8.7)<br>2/23 (8.7)<br>4/23 (17.4)<br>2/23 (8.7)<br>2/23 (8.7)<br>1/23 (4.3)<br>10/23 (43.5)	 2/8 (25.0)<br>0<br>0<br>1/8 (12.5)<br>2/8 (25.0)<br>0<br>3/8 (37.5)	 0<br>2/14 (14.3)<br>4/14 (28.6)<br>1/14 (7.1)<br>0<br>1/14 (7.1)<br>6/14 (42.9)	0.082
mRS at 3 months 0-1 (%)	4/23 (17.4)	2/8 (25.0)	2/14 (14.3)	0.602
mRS at 3 months 0-2 (%)	8/23 (34.8)	2/8 (25.0)	6/14 (42.9)	0.649
mRS at 3 months 0-3 (%)	10/23 (43.5)	3/8 (37.5)	7/14 (50.0)	0.675
mRS at 3 months 2-6 (%)	19/23 (82.6)	6/8 (75.0)	12/14 (85.7)	0.602
mRS at 3 months 3-6 (%)	15/23 (65.2)	6/8 (75.0)	8/14 (57.1)	0.649
mRS at 3 months 4-6 (%)	13/23 (56.5)	5/8 (62.5)	7/14 (50.0)	0.675
Intrahospital death (%)	10 (34.5)	3 (25.0)	6 (37.5)	0.687
Death at 3 months (%)	10/23 (43.5)	3/8 (37.5)	6/14 (42.9)	1.0

mRS= modified Rankin Scale

Supplementary Table S3. Plasma levels of molecular biomarkers at various timepoints (T0, T1, T2) in all study patients and by presence/absence of ischemic lesions at T1 (statistically significant or borderline statistically significant differences are in bold)

	**All patients N=28**	**Ischemic lesions n=12**	**No ischemic lesions n=16**	**p value**
NO, µM, mean (IQR)				
- T0	18.42 (8.68-28.62)	15.02 (6.35-27.40)	19.71 (9.0-30.58)	0.386
- T1	22.76 (19.04-42.66)	20.69 (18.45-39.0)	29.40 (17.03-44.69)	0.749
- T2	28.39 (15.35-48.34)	28.52 (14.89-48.34)	28.08 (16.44-65.53)	1.0
ET-1, pg/mL, mean (IQR)				
- T0	18.74 (14.05-22.12)	19.92 (17.19-33.43)	16.40 (11.38-20.22)	0.110
- T1	15.71 (12.46-21.62)	18.67 (11.81-26.15)	13.66 (13.02-20.29)	0.630
- T2	13.96 (12.15-17.63)	15.07 (11.91-17.63)	13.65 (10.78-19.51)	0.831
sNOX2-dp, pg/mL mean (IQR)				
- T0	27.51 (18.91-37.30)	34.94 (27.96-42.0)	22.40 (18.37-27.75)	**0.051**
- T1	14.50 (9.41-19.79)	17.50 (8.59-20.92)	11.57 (9.52-17.87)	0.749
- T2	18.73 (12.07-24.20)	18.99 (14.50-24.20)	14.84 (4.34-20.43)	0.522
ADMA, ng/mL, mean (IQR)				
- T0	128.70 (111.73-169.43)	167.94 (119.43-175.07)	121.89 (103.67-146.92)	0.131
- T1	110.69 (98.78-120.33)	99.44 (94.28-116.27)	116.55 (106.67-146.20)	0.078
- T2	116.56 (92.06-126.77)	116.56 (97.20-126.34)	107.56 (91.97-144.70)	0.831

NO= nitric oxide; IQR= Interquartile range; ET-1= endothelin 1; sNOX2-dp = soluble NOX2-derived peptide; ADMA= asymmetric dimethyl-arginine.

Supplementary Table S4. Median changes in plasma levels of molecular biomarkers over time in all study patients and by presence/absence of ischemic lesions at T1 (statistically significant differences are in bold)

	**All patients N=28**	**Ischemic lesions n=12**	**No ischemic lesions n=16**	**p value**
NO, μM, mean (IQR)				
- ΔT1-T0	9.97 (-8.86, 20.79)	9.54 (-1.88, 16.46)	20.79 (-9.11, 29.27)	0.361
- ΔT2-T1	5.96 (-14.33, 24.90)	9.23 (-11.21, 24.90)	-3.82 (-18.78, 24.82)	0.670
- ΔT2-T0	20.62 (-7.24, 29.88)	21.13 (-21.30, 0.78)	11.81 (-3.72, 46.63)	0.831
ET-1, pg/mL, mean (IQR)				
- ΔT1-T0	-0.23 (-6.66, 2.53)	-3.16 (-12.63, 3.66)	-1.09 (-5.59, 2.27)	0.631
- ΔT2-T1	-2.08 (-8.28, 1.58)	-3.93 (-10.26, 1.58)	-1.05 (-6.18, 5.50)	0.394
- ΔT2-T0	-2.62 (-12.60, 0.84)	-6.89 (-21.30, 0.78)	-0.34 (-4.61, 1.56)	0.136
sNOX2-dp, pg/mL, mean (IQR)				
- ΔT1-T0	-13.32 (-19.28, -4.60)	-18.56 (-23.78, -12.37)	-7.24 (-12.18, -2.31)	**0.037**
- ΔT2-T1	-1.83 (-3.82, 16.47)	-1.83 (-3.70, 16.47)	0.13 (-5.98, 19.21)	0.831
- ΔT2-T0	- 17.66 (-20.70, 0.22)	-18.77 (-23.12, -5.19)	-10.90 (-18.59, 14.56)	0.286
ADMA, ng/mL, mean (IQR)				
- ΔT1-T0	-30.72 (-45.20, -6.42)	-38.04 (-61.38, -21.45)	-16.27 (-34.24, 45.25)	0.150
- ΔT2-T1	-7.15 (-19.88, 31.84)	10.86 (-16.20, 31.84)	-18.46 (-25.52, 30.33)	0.394
- ΔT2-T0	-37.02 (-54.56, 10.39)	-49.86 (-58.58, 10.39)	-13.65 (-47.13, 22.44)	0.286

NO= nitric oxide; IQR= Interquartile range; ET-1= endothelin 1; sNOX2-dp = soluble NOX2-derived peptide; ADMA= asymmetric dimethyl-arginine.

A.

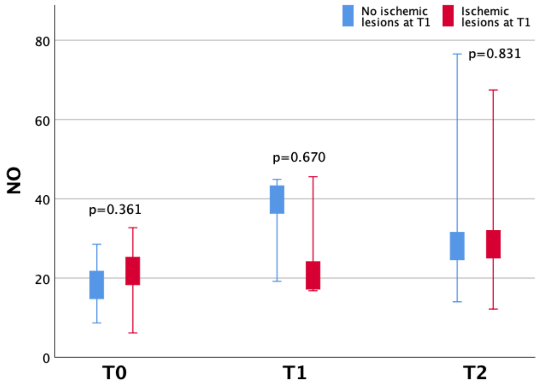

B.

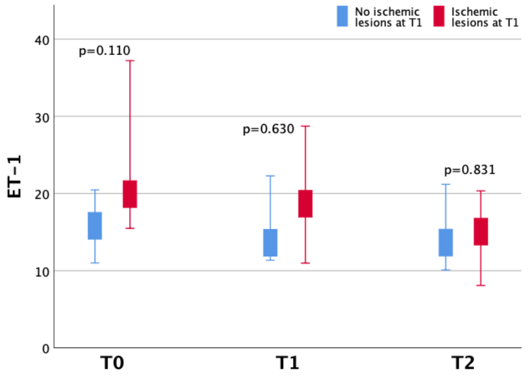

C.

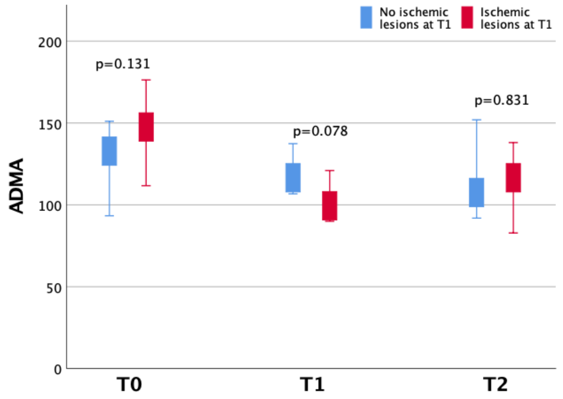

Supplementary Figure S1. Median plasma levels of molecular biomarkers at different timepoints (T0, T1, T2) by presence/absence of ischemic lesions at T1. (**A**) NO (plasma levels in μM); (**B**) ET-1 (plasma levels in pg/mL); (**C**) ADMA (plasma levels in ng/mL).

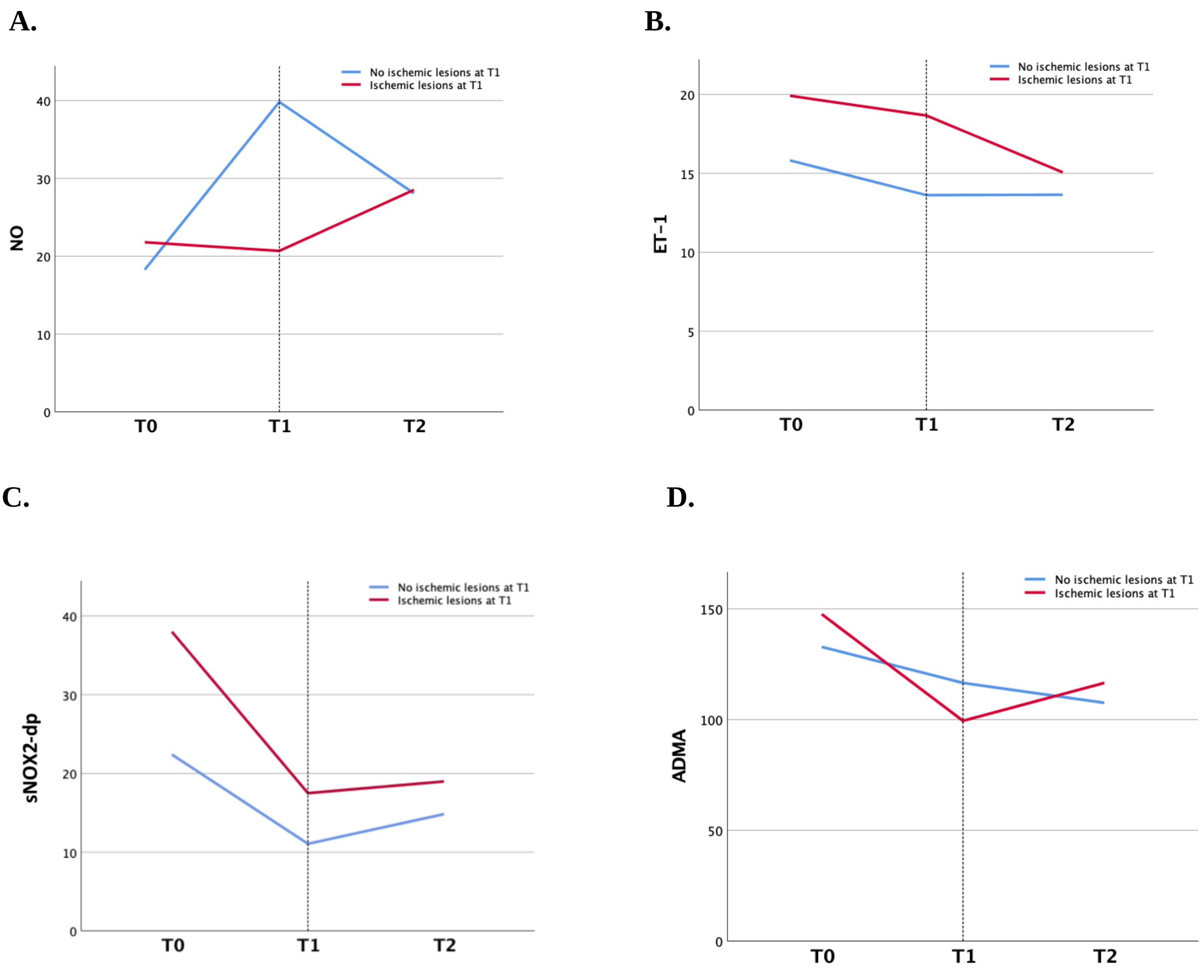

Supplementary Figure S2. Time profile of median plasma levels of molecular biomarkers at different timepoints by presence/absence of ischemic lesions at T1. (**A**) NO (plasma levels in µM); (**B**) ET-1 (plasma levels in pg/mL); (**C**) sNOX2-dp (plasma levels in pg/mL); (**D**) ADMA (plasma levels in ng/mL).

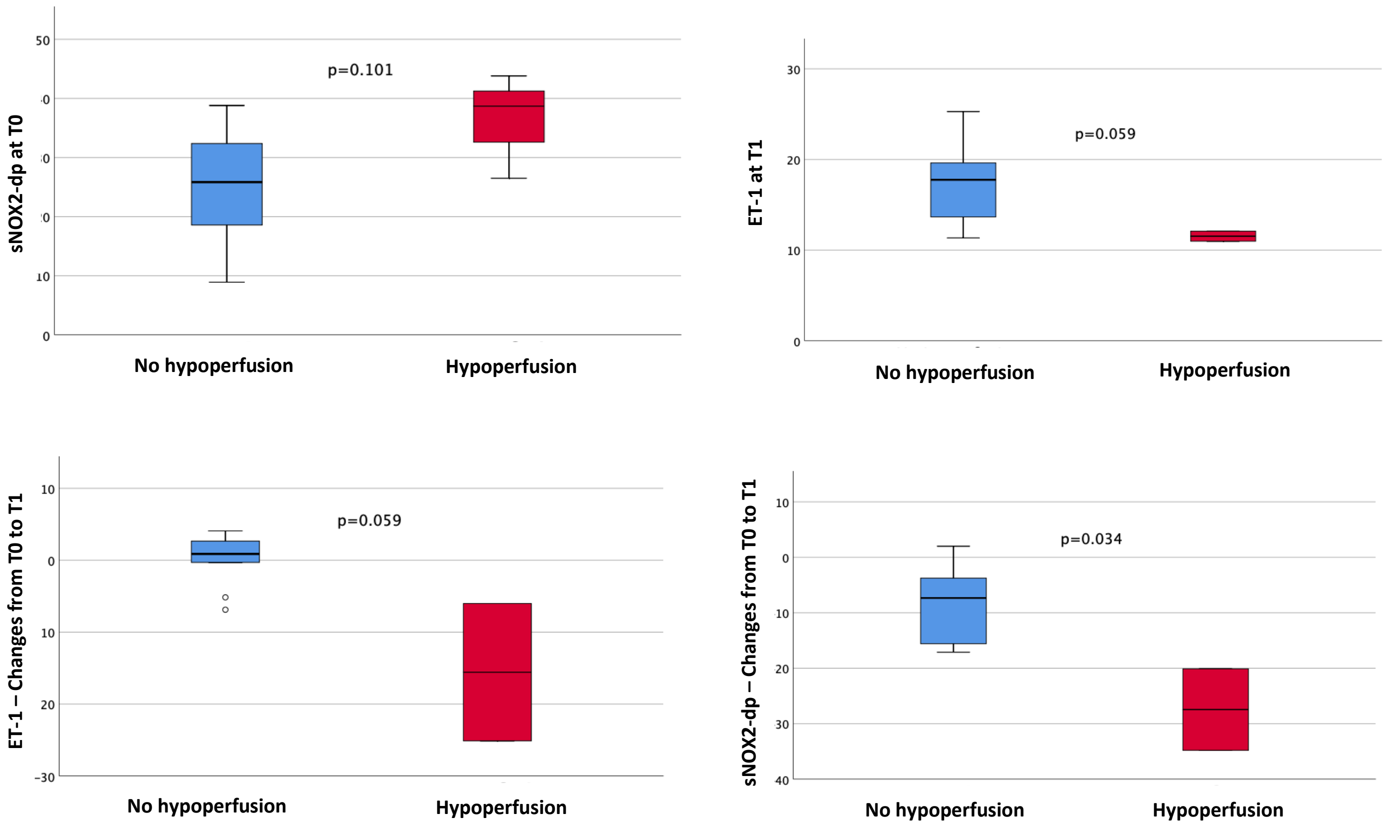

	No hypoperfusion (n=14)	Hypoperfusion (n=3)	p value
sNOX2-dp at T0, pg/mL, median (min, max)	25.85 (8.91, 38.80)	38.69 (26.49, 43.81)	0.101
ET-1 at T1, pg/mL, median (min, max)	17.76 (11.35, 25.29)	11.54 (10.98, 12.09)	0.059
ET-1 T0-T1, pg/mL, median (min, max)	0.88 (-6.88, 4.08)	-15.57 (-25.12, -6.01)	0.059
sNOX2-dp T0-T1, pg/mL, median (min, max)	-7.33 (-17.11, 2.0)	-27.45 (-34.79, -20.11)	0.034

sNOX2-dp = soluble NOX2-derived peptide; ET-1= endothelin 1.

Supplementary Figure S3. Associations between molecular biomarkers and hemispheric hypoperfusion at T1.

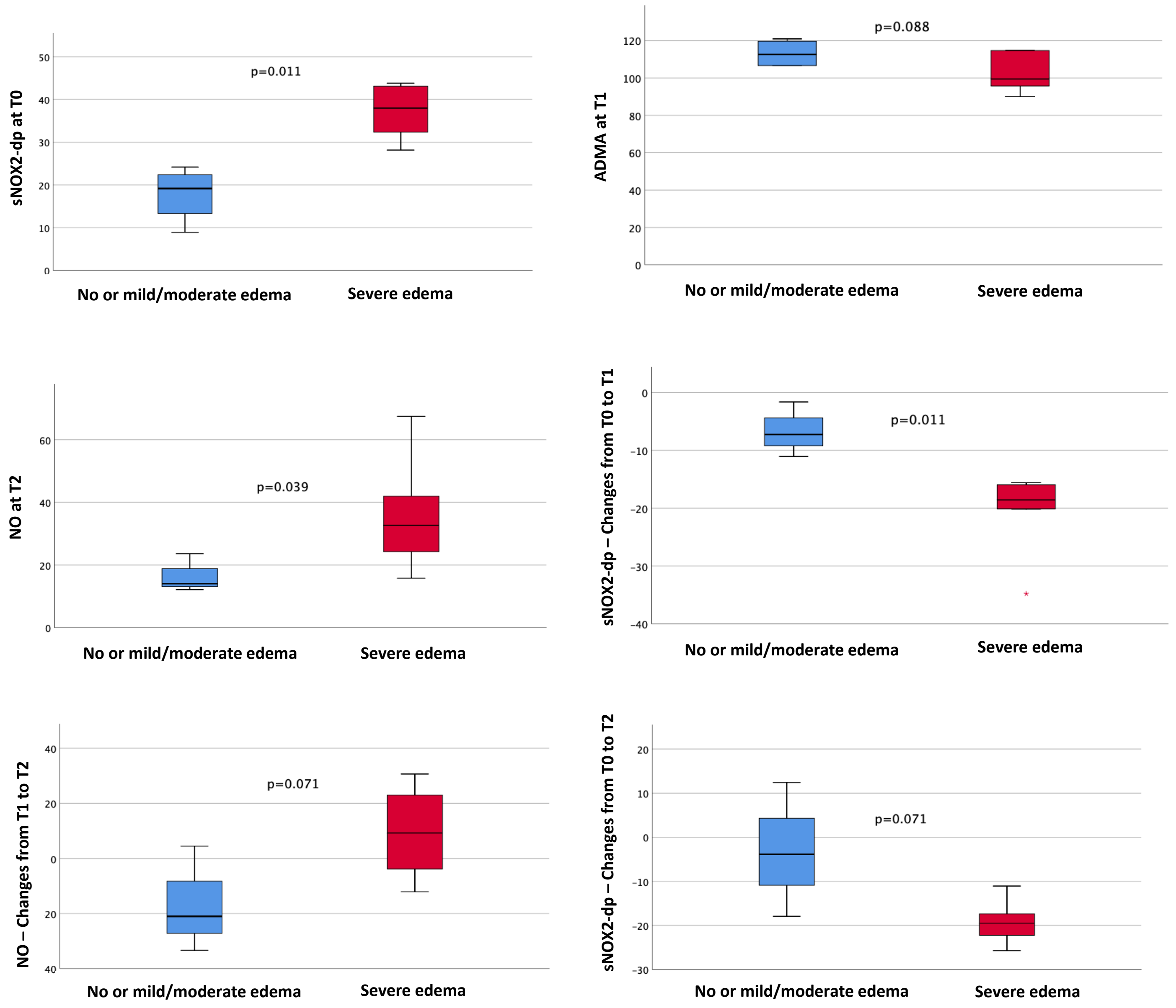

	**No or mild-moderate edema (n=4)**	**Severe edema (n=6)**	**p value**
sNOX2-dp at T0, pg/mL, median (min, max)	19.17 (11.11, 23.29)	37.99 (31.32, 43.28)	0.011
ADMA at T1, ng/mL, median (min, max)	112.57 (106.59, 120.33)	99.44 (94.28, 114.68)	0.088
NO at T2, μM, median (min, max)	14.03 (12.18, …)	32.64 (22.15, 48.34)	0.039
sNOX2-dp T0-T1, pg/mL, median (min, max)	-7.24 (-10.12, -2.99)	-18.56 (-23.78, -15.86)	0.011
NO T1-T2, μM, median (min, max)	-21.0 (-33.38, …)	9.23 (-5.89, 24.90)	0.071
sNOX2-dp T0-T2, pg/ml, median (min, max)	-3.12 (-17.95, …)	-19.49 (-23.12, -15.79)	0.071

sNOX2-dp = soluble NOX2-derived peptide; ADMA= asymmetric dimethyl-arginine; NO= nitric oxide.

Supplementary Figure S4. Associations between molecular biomarkers and severity of brain edema at T2.

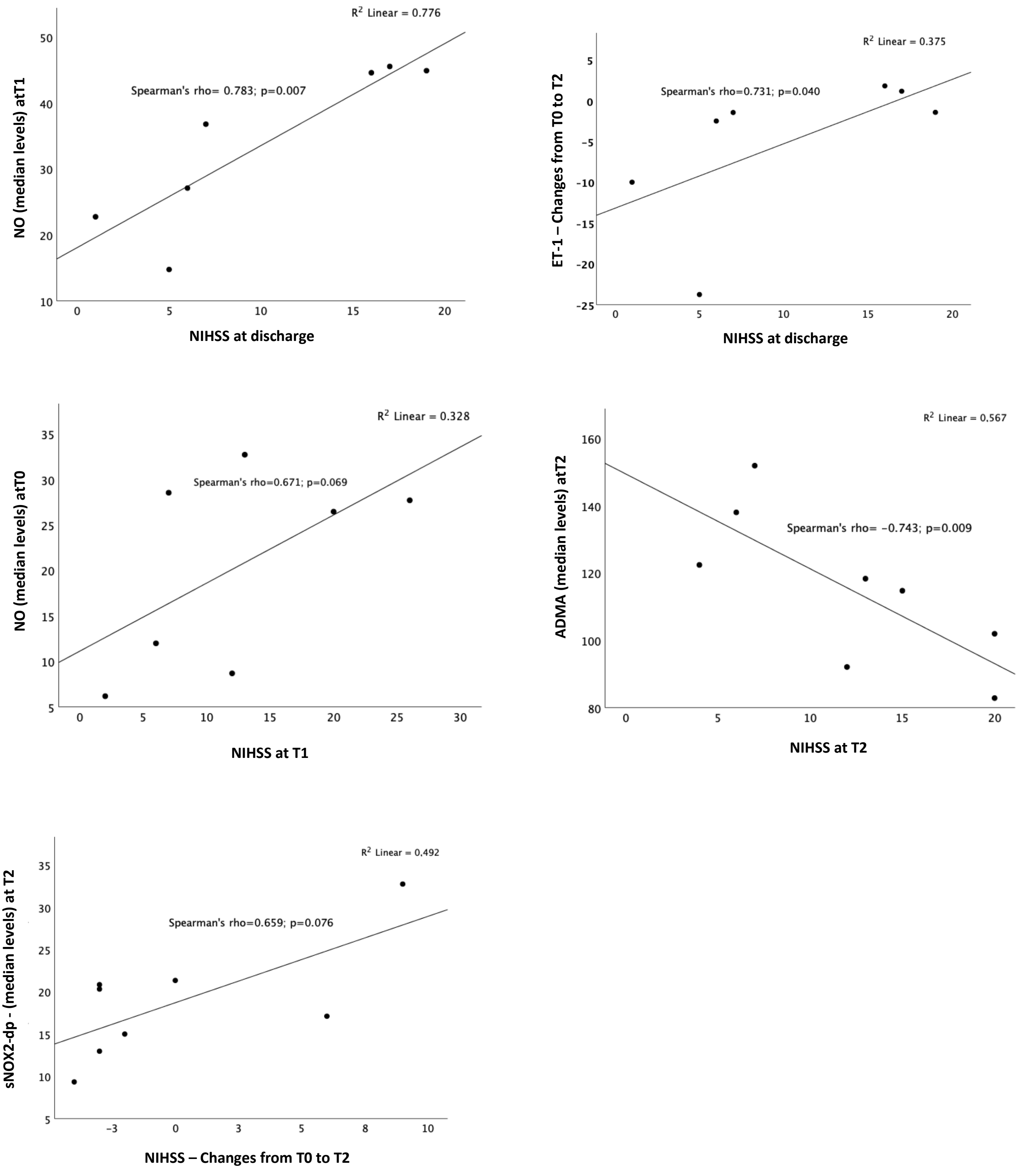

Supplementary Figure S5. Correlations between molecular biomarkers and NIHSS.

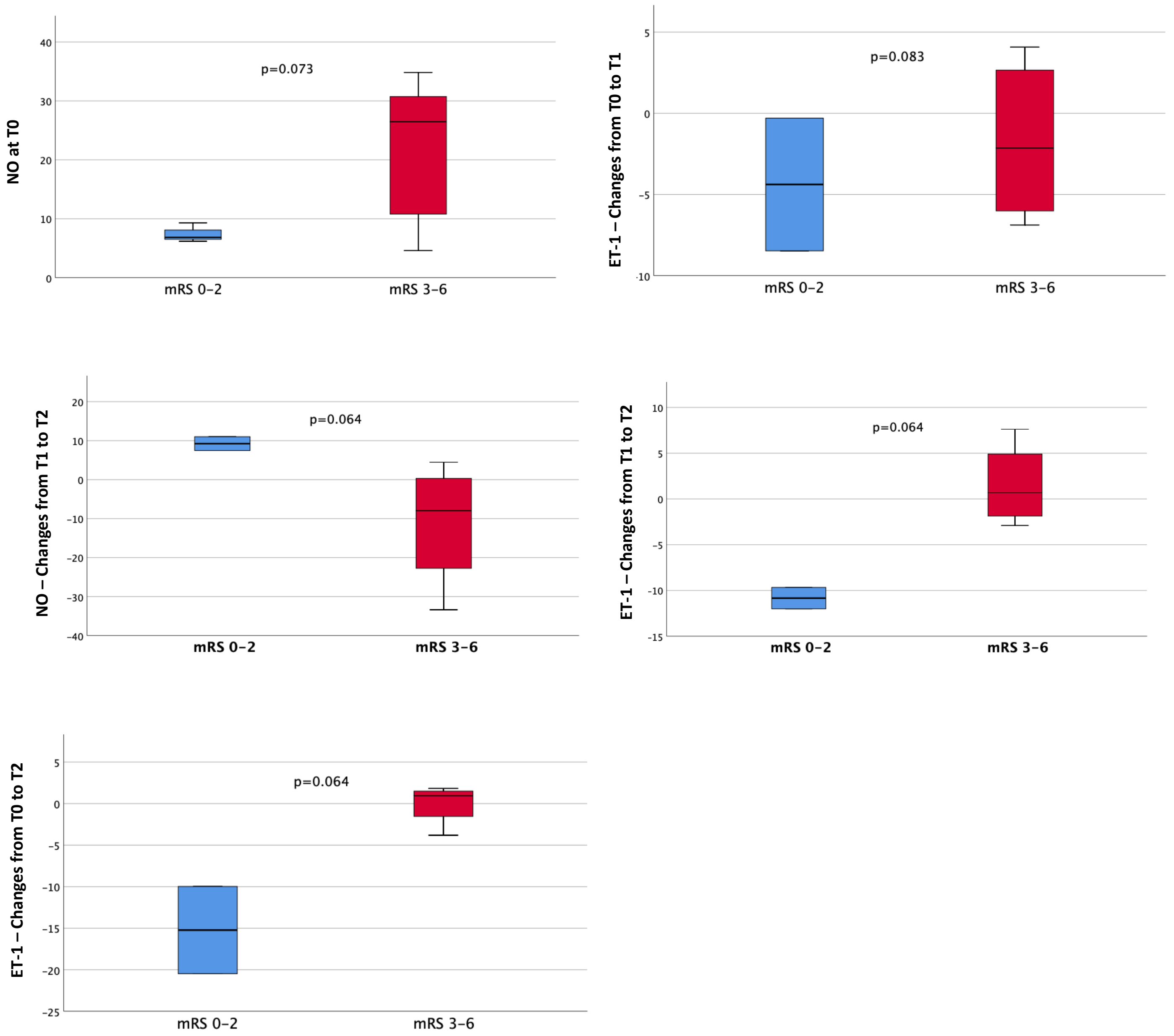

	mRS 0-2 (n=3)	mRS 3-6 (n=3)	p value
NO at T0, median (min, max)	6.85 (6.18, 9.31)	26.46 (34.82, 8.69)	0.073
ET-1 at T0, pg/mL, median (min, max)	29.17 (18.06, 37.20)	17.38 (8.56, 34.83)	0.083
NO T1-T2, µM, median (min, max)	9.23 (7.45, 11.01)	-7.96 (-33.38, 4.45)	0.064
ET-1 T1-T2, pg/mL, median (min, max)	-10.84 (-12.01, -9.67)	0.69 (-2.90, 7.61)	0.064
ET-1 T0-T2, pg/mL, median (min, max)	-15.23 (-20,48, -15.23)	0.96 (-3.81, 1.83)	0.064

mRS=modified Rankin Scale; NO= nitric oxide; ET-1= endothelin 1.

Supplementary Figure S6. Associations between molecular biomarkers and clinical outcome at 3 months.

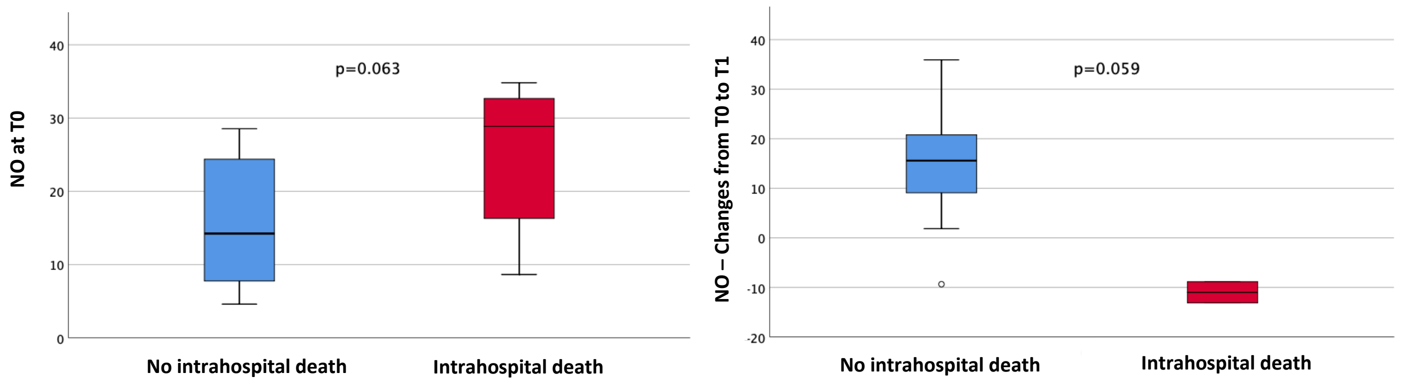

	No intrahospital death (n=11)	Intrahospital death (n=8)	P value
NO at T0, μM, median (min, max)	14.24 (28.54, 28.54)	28.86 (8.65, 34.82)	0.063
NO T0-T1, μM, median (min, max)	15.58 (807.81, 2407.32)	-10.99 (-13.11, -8.86)	0.059

NO= nitric oxide.

Supplementary Figure S7. Associations between molecular biomarkers intrahospital death.

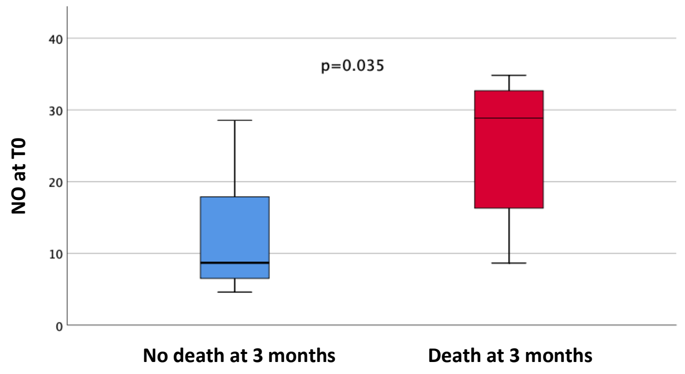

	No death at 3 months (n=7)	Death at 3 months (n=8)	p value
NO at T0, μM, median (min, max)	8.69 (4.61, 28.54)	28.86 (8.65, 34.82)	0.035

NO= nitric oxide.

Supplementary Figure S8. Associations between molecular biomarkers and death at 3 months.
